# Dr. Walker’s Modification of Sayre’s Apparatus

**Published:** 1879-01

**Authors:** 


Dr. Walker's Modification of Sayre's Apparatus.—Judging from the discussion by the Medical Society, the surgeons of this country are willing to adopt the treatment of Dr. Sayre for disease of the spine in its entirety. Many, however, who have become acquainted with Dr. Walker's mode of applying the plaster jacket will think, with me, that it has some merits over that of Dr. Sayre, and I wish briefly to record in the Journal my own experience of this modification, and my reasons for adopting it.

At the Children's Hospital, and other institutions, I have had a very great number of cases of diseased spine under my care; and, until August last, when, as Secretary to the Surgical Section, at the meeting of the British Medical Association, I had full opportunities of learning Dr. Walker's views, I had always applied the plaster bandage with strict attention to all the rules laid down by Dr. Sayre. After conquering those small technical difficulties which beset the adoption of any new plan of treatment, I found that, apart from the terror, often the pain, and sometimes the danger of Dr. Sayre's method, it was impossible to avoid, in some cases, the occurrence of sores over the prominent spinous processes of more severe cases.

Since August I have applied, at Great Ormond Street and elsewhere, more than twenty bandages after the method advocated by Dr. Walker, and I confess myself most satisfied with the result in every way. In all slighter cases the effect has been as satisfactory as Dr. Sayre himself could wish to see; whilst, of the more severe cases, in no instance have I had to remove the jacket. In application it is simple, and it is free from any terrors, pains, or dangers, which undoubtedly are caused by suspension of the patient. On the other hand, it is most efficacious in result, and based on the soundest principles, pathological and surgical.

The various elements of the spine are held together by ligaments, whose function is to fix each vertebra in a definite relation to the vertebra above and below, the relations varying in different parts of the spine, but the whole being subservient to maintaining the superincumbent weight in the erect position. If by the collapse of the body of one or more vertebræ the weight be thrown forward, a strain is put upon the muscles and ligaments so long as that weight has to be supported; but, whenever the weight is removed, the vertebræ return to their normal relationships. The method which Dr. Sayre adopts of removing the weight is by suspending the patient; that recommended by Dr Walker is by laying the patient in the recumbent position. English surgeons appear to be so carried away by the novelty of ruspending patients by the head and axillæ, that they are unwilling to believe that the same result can be obtained by laying them on their back. Whilst the one is an unnatural means of effecting the object, not devoid of risk or free from pain, the other is an easy method, and that which is apparently dictated by nature. It must not be forgotten that, before Dr. Sayre came amongst us, many a case of angular curvature was brought to a successful issue by rest long continued in the recumbent position. To Dr. Sayre is undoubtedly due all credit for discovering that the spine could be fixed in a condition of rest and treated in the way that experience proves to be most useful in the case of disease in other joints. But by the plan of Dr. Walker the vertebræ are fixed in that condition which they assume when the patient is laid upon the back, the position of most natural rest. In principle this seems to me to be scientific; in practice it is simple; and in result it is efficacious.

I shall be happy to show the mode which I have adopted, after Dr. Walker, of treating these cases at the Children's Hospital, and I hope that it may commend itself to others as producing an equally beneficial result, whilst avoiding the many objections which are found to suspension.—*John H. Morgan in British Medical Journal.*

